# Circular Permutation in the Ω-Loop of TEM-1 β-Lactamase Results in Improved Activity and Altered Substrate Specificity

**DOI:** 10.1371/journal.pone.0035998

**Published:** 2012-04-19

**Authors:** Gurkan Guntas, Manu Kanwar, Marc Ostermeier

**Affiliations:** Department of Chemical and Biomolecular Engineering, Johns Hopkins University, Baltimore, Maryland, United States of America; Center for Genomic Regulation, Spain

## Abstract

Generating diverse protein libraries that contain improved variants at a sufficiently high frequency is critical for improving the properties of proteins using directed evolution. Many studies have illustrated how random mutagenesis, cassette mutagenesis, DNA shuffling and similar approaches are effective diversity generating methods for directed evolution. Very few studies have explored random circular permutation, the intramolecular relocation of the N- and C-termini of a protein, as a diversity-generating step for directed evolution. We subjected a library of random circular permutations of TEM-1 β-lactamase to selections on increasing concentrations of a variety of β-lactam antibiotics including cefotaxime. We identified two circularly permuted variants that conferred elevated resistance to cefotaxime but decreased resistance to other antibiotics. These variants were circularly permuted in the Ω-loop proximal to the active site. Remarkably, one variant was circularly permuted such that the key catalytic residue Glu166 was located at the N-terminus of the mature protein.

## Introduction

Directed evolution is a powerful technique used to improve protein properties. Directed evolution involves the generation of a protein library and subsequent rounds of selection or screening to identify improved protein variants. Generation of diverse protein libraries containing improved variants is therefore important for successful application of directed evolution techniques. Many studies have demonstrated the use of random mutagenesis, cassette mutagenesis, DNA shuffling and similar approaches as effective diversity generating methods for directed evolution. Circular permutation is the intramolecular relocation of the N- and C-termini of a protein [1]. Circular permutation is an atypical method for diversity generation, since it can be used to change the linear order of the primary sequence, but not the identity of the amino acids in the sequence. Circular permutation can alter the folding kinetics of a protein and create variants that could retain comparable wild-type functionality [2], [3], [4], [5].

Very few directed evolution studies have used circular permutation as the diversity-generating step. However, there is growing evidence to suggest that the approach has merit for certain applications, including improving enzyme activity and altering substrate specificity. Although the goal was not to improve enzyme activity, a few circular permuted 5-aminolevulinate synthases were found to have higher catalytic efficiencies than the wild-type enzyme [6]. More recently, random circular permutation has been successfully applied as the diversity generation step for the directed evolution of *Candida antarctica* lipase B [7], [8] and *Bacillus circulans* xylanase Bcx [9]. Circular permutation at select sites in lipase B was found to increase hydrolytic activity up to 175-fold on certain substrates [8], [10]. Such improved variants often have the relocated termini proximal to the active site [7], [9], which may alter activity through local, subtle conformational changes and increased backbone flexibility [11]. Circular permutation has also found use in directed evolution for creating diversity in the fusion geometry between two proteins in order to create protein switches [12], [13].

TEM-1 β-lactamase (BLA) is a class A periplasmic bacterial enzyme that can hydrolyze a wide range of penicillin and cephalosporin antibiotics by a mechanism that involves the acylation and deacylation of a serine residue [14]. BLA cleaves the four atom β-lactam ring and deactivates the antibacterial properties of the antibiotic. This renders the antibiotics ineffective against gram-negative bacteria like *E. coli*. BLA's Ω-loop is considered to play an important role in the catalytic activity of BLA against β-lactam antibiotics [15], [16], [17]. The relative proximity of BLA's N- and C-termini makes the enzyme a good candidate for circular permutation; however, rationally designed circular permutants were found to be poor catalysts compared to the wild type protein [18].

We previously reported the creation of a hybrid of maltose binding protein (MBP) and BLA in which BLA was circularly permuted in the Ω-loop and inserted into MBP [19]. TEM-1 β-lactamase has poor activity against third-generation cephalosporin β-lactams such as cefotaxime (CFTX) in part due to steric reasons; however, many substitutions within and outside the Ω-loop are known to increase CFTX resistance and some of which are believed to act by repositioning the Ω-loop and enlarging the active site cavity [17]. The MBP-BLA hybrid gene conferred wild-type resistance to CFTX but reduced resistance to other β-lactam antibiotics. We speculated that the relatively high resistance to CFTX was a result of the circular permutation of BLA in the Ω-loop, since a change in substrate specificity due to mutations and insertions in or near the Ω-loop has been observed [15], [16], [17]. This result led us to wonder how effective circular permutation would be for altering the catalytic activities of BLA. We present here the results of a study to address this question.

## Results and Discussion

### Circular permutation of BLA and insertion into MBP alters BLA substrate specificity

We have previously used random circular permutation as a diversity-generating tool for the creation of protein switches. An iterative process involving random circular permutations of *bla* and random insertions of *bla* into the gene for maltose binding protein yielded hybrid proteins in which BLA enzyme activity was modulated by maltose [19]. One particular switch, MBP317-347, was identified from Library 7 in which random circular permutations of *bla* were inserted in place of the codon 317 in the gene for MBP. MBP317-347 has severely compromised catalytic activity in the absence of maltose that increases several hundred-fold in the presence of maltose. However, the catalytic activity in the presence of maltose was 40-fold less than wild-type BLA. Consistent with this, *E. coli* cells expressing MBP317-347 had a lower minimum inhibitory concentrations (MIC) for a variety of penicillins and cephalosporins than did cells expressing BLA (about 8–16 fold lower; Table 1). However, the MIC for cefotaxime for cells expressing either MBP317-347 or BLA were the same, suggesting that MBP317-347's substrate specificity was different than BLA and potentially, that MBP317-347 had higher activity than BLA for hydrolyzing cefotaxime.

**Table 1:** MIC of beta-lactam antibiotics conferred by BLA and variants of BLA.

MIC for indicated antibiotic (µg/ml)				
Protein	Cefotaxime	Ampicillin	Cefazolin	Cephalothin
**MBP** a **(Control)**	0.02	2	1	4
**MBP317-347** a	0.04	512	2	8
**BLA** b	0.04	8192	16	64
**CFX011** b	0.18	≤2048	≤8	≤32
**CFX019** b	0.16	≤2048	≤8	≤32

a at 37°C.

b at 22°C.

### Random circular permutation of BLA

We wondered if the altered catalytic activity of the BLA domain of MBP317-347 might be a result of the circular permutation. The BLA domain in MBP317-347 is circular permuted within the Ω-loop that contains the key active site residue Glu166. Alternatively, the altered specificity could be a result of perturbations resulting from insertion of the BLA domain into MBP. We also wondered if circular permutation might be a route for improving enzyme activity in general. Systematic circular permutation and kinetic characterization of dihydrofolate reductase had earlier identified a few circular permutants with modest improvements in catalytic activity [5]. However, at the time we began these experiments it was unknown to what extent random circular permutation employed as a diversity-generating step in directed evolution would result in gain of function mutants, since such an experiment had not been reported. We thus examined whether random circular permutation of *bla* would result in enzymes with improved catalytic activity. Qian and Lutz have since demonstrated how random circular permutation employed for the directed evolution of *Candida antartica* lipase B can produce substantial improvements (up to 175-fold) in catalytic activity on some substrates [7], [8].

### Construction and characterization of the library of circularly permuted BLA

**Figure 1:**
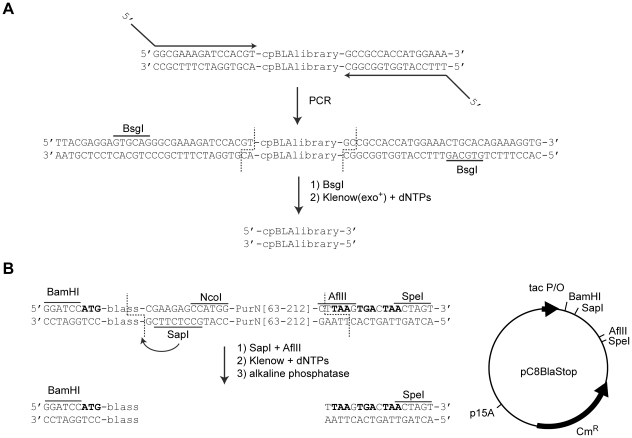
Library construction. (A) The previously described collection of circularly permuted *bla* genes [19] was PCR-amplified using primers designed to anneal just outside the circularly permuted *bla* DNA. Both primers contained an appropriately spaced BsgI restriction site (cuts and the indicated dashed lines) such that treatment of the digested product to remove the two-base 3′ overhand would result in the circularly permuted *bla* library without any “scars” from the surrounding DNA. (B) Plasmid pC8BlaStop is derived from pDIM-C8 [23] and contains appropriately place SapI and AflII sites such that fusion of the circularly permuted *bla* library can occur seamlessly to the *bla* signal sequence (blass) and a series of stop codons in all three reading frames (in bold).

The library of random circular permutations of *bla* contained in Library 7 [19] was amplified using primers designed to anneal to the MBP gene on either side of *bla*. The primers had BsgI sites such that digestion of the PCR product with BsgI followed by degradation of the 3′ overhangs by Klenow would result in a blunt ended product with no DNA originating from the MBP gene (Figure 1A). The original N- and C-termini of BLA are joined by a DKS linker in this library. The DKS linker has been previously identified as a beneficial tri-peptide linker for BLA circularly permuted at residue 216 [18]. The circularly permuted *bla* gene library was inserted into a vector such that it was fused to a sequence coding for the natural 23 amino acid *bla* signal sequence on the 5′ end and a series of three stop codons in all reading frames at the 3′ end (Figure 1B). The signal sequence is necessary to export BLA to the periplasm and to confer significant resistance to β-lactam containing antibiotics.

The number of transformants in the library that had a single insert was 5×10^5^. This greatly exceeds the number of possible “perfect” circular permutations of the gene (798). However, the process of random circular permutation results in tandem duplications and deletions at the site of circular permutation in addition to perfect circular permutations. In addition, the insert can be either inserted in the correct orientation, or in the reverse orientation. If one estimates that at each position there could also be deletions and duplications of up to 50 bp each, the theoretical degeneracy of the library is 798×(50+50)×2 = 1.596×10^5^. Assuming all variants are equally probable, the probability that our library contains the most active member of these possible variants is 87% [20].

**Figure 2:**
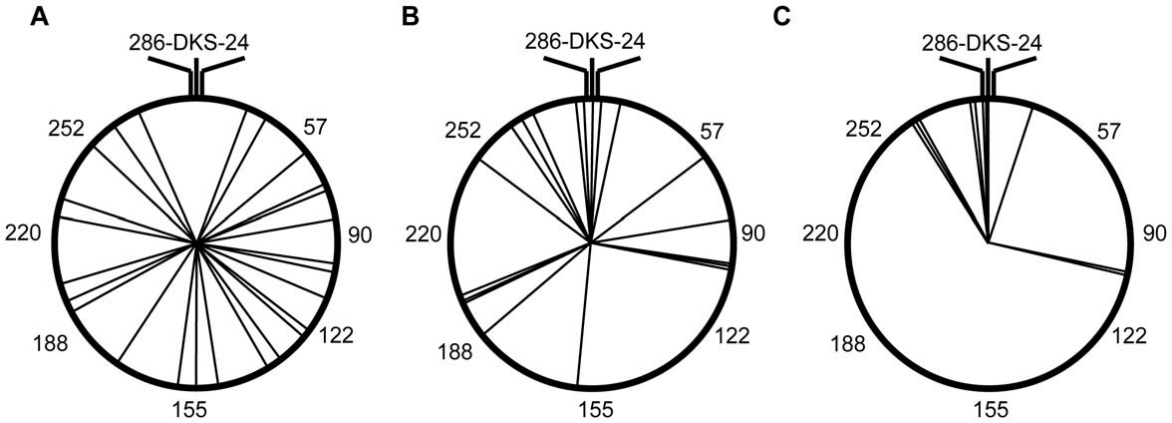
Sites of circular permutation BLA based on DNA sequencing of (A) 25 randomly selected members from the naïve library, (B) 20 randomly selected members capable of growing on plates containing 16 µg/ml ampicillin, and (C) 10 randomly selected members capable of growing on plates containing 250 µg/ml ampicillin. The first amino acid in the mature wild-type BLA is 24 (since the signal sequence of amino acids 1–23 is removed) and the last amino acid is 286. The linker joining the N- and C-termini has the amino acid sequence DKS.

Sequencing of 25 random members of the naive library indicated no obvious bias in the site of circular permutation (Figure 2A). Next, functional library members were selected by plating the naïve library on LB agar plates supplemented with 16 µg/ml ampicillin (this concentration is 8-fold higher than the MIC for cells not expressing any β-lactamase) or with 250 µg/ml ampicillin. Functional variants from each plating condition were chosen at random and sequenced (Figure 2B&C). Active variants identified from the 250 µg/ml ampicillin plates were heavily biased towards circular permutations very near the original N- and C-termini of the protein (Figure 2C). The site of circular permutation was more varied for those clones selected at the lower ampicillin concentration (Figure 2B).

### Selection of BLA variants that conferred improved antibiotic resistance

The above selection was for minimally functional β-lactamase activity. Selections were subsequently performed on the naïve library to identify library members that exhibited antibiotic resistance above that conferred by *bla*. The naïve library was plated (both at 37°C and at room temperature) on plates containing one of four antibiotics (ampicillin, cefazolin, cephalothin and cefotaxime) at or above the MIC for these antibiotics conferred by *bla*. Only in the case of cefotaxime were clones with elevated resistance identified. The number of colonies that grew indicated that about three library members exhibited resistance to at least 0.08 µg/ml cefotaxime and room temperature. The plasmid DNA from fourteen colonies that grew at room temperature on agar plates supplemented with 0.08 or 0.10 µg/ml cefotaxime were isolated and retransformed into fresh *E. coli* cells. Six colonies were false positives since the retransformed cells did not have resistance above background. The remaining eight library clones had an MIC for cefotaxime of at least 0.12 µg/ml (3-fold higher than the MIC of cells expressing BLA).

Sequencing revealed two library members with very similar sequences: CFX011 (BLA[166-286]-DKS-BLA[24-163]-VL) and CFX019 (BLA[157-286]-DKS-BLA[24-163]-VL). Like the BLA domain of MBP317-347, these two variants are circularly permuted in the Ω-loop. This change in substrate specificity due to alteration in or near the Ω-loop is consistent with previous reports [15], [16]. Interestingly, CFX011 is permuted at exactly Glu166, which is known to be a key catalytic residue [21].

### Characterization of the improved circularly permuted variants

The MIC conferred by *CFX011* and *CFX019* were determined on a number of antibiotics and compared to that exhibited by *bla* (Table 1). Whereas the MIC for cefotaxime were 4-fold higher, the MIC's for ampicillin, cephalothin and cefazolin were all considerably lower than that conferred by *bla*. This indicates that the increased level of resistance provided by these two variants cannot solely be for reasons of increased expression of the protein, and that circular permutation has resulted in an alteration of the substrate specificity of the enzyme. Experiments designed to express CFX011 and CFX019 using the vectors on which they were isolated suffered from poor expression of the circular permutants (as judged by western blots and β-lactamase activity assays on lysates) and poor yields upon attempted purification using a phenylboronic acid agarose affinity column. This suggests that circular permutation compromised the production of CFX011 and CFX019 in *E. coli*. Thus, the most likely explanation for the increase MIC conferred by *CFX011* and *CFX019* is increased specific activity for hydrolyzing cefotaxime. Alternatively, these variants would have to be expressed at significantly higher level than BLA, retain TEM-1 levels of activity on cefotaxime, and selectively lose activity on ampicillin, cephalothin and cefazolin. This scenario seems unlikely.

### Conclusions

A directed evolution strategy involving random circular permutation was successfully applied to increasing the ability of TEM-1 β-lactamase to provide resistance to the antibiotic cefotaxime. The genes that provided increased resistance to CFTX were circularly permuted in the region corresponding to the Ω-loop of the protein. The genes conferred decreased activity against other cephalosporins and ampicillin. This finding indicates that circular permutation altered the substrate specificity of the enzyme. Since increased expression of the circularly permuted variants relative to wild-type would be unlikely, we proposed that the increased resistance derives from an increase in catalytic activity on cefotaxime, although confirmation will require characterization of the catalytic activity in vitro. It is interesting that insertions in the Ω-loop generated by pentapeptide mutagenesis also resulted in increased resistance to CFTX [15], indicating that different perturbations in this loop can result in similar changes in specificity, perhaps by removing steric constraints.

## Materials and Methods

### Materials

All enzymes were purchased from New England Biolabs. All DNA purification kits were purchased from Qiagen. Cefotaxime and ampicillin were purchased from Fisher Scientific. All oligonucleotides were purchased from Invitrogen. pGEM T-vector cloning kit and Taq polymerase were purchased from Promega (Madison, WI). All other chemicals were purchased from Sigma.

### Circular permutation library construction

The library was constructed in plasmid pC8BlaStop, a plasmid derived from pDIM-C8 [22] in which the section between the BamHI and SpeI sites is modified according to Figure 1A. Ten µg of pC8BlaStop was digested with 36 units of *AflII* and 25 units of *SapI*. The desalted digestion product was treated with Klenow and alkaline phosphatase according to the manufacturer's specifications. The large blunt fragment was isolated by agarose gel electrophoresis.

The insert was derived from a previously described library (called Library 7) of circularly permuted *bla* genes that was inserted into a specific location in the gene encoding maltose binding protein [19]. To create the library for the work described here we amplified this library of circularly permuted *bla* genes by PCR using the following primers: 5′-TTACGAGGAGTGCAGGGCGAAAGATCCACGT-3′ and 5′-CACCTTTCTGTGCAGTTTCCATGGTGGCGGC-3′ using 40 ng of template DNA. These primers located flanking BsgI sites an appropriate distance from the circularly permuted library such that digestion of the PCR product with BsgI and treatment with Klenow and dNTPs produced a blunt ended product without any extra DNA from the *malE* gene (Figure 1B). From the PCR reaction DNA of the correct size was isolated by agarose gel electrophoresis. Five µg of isolated DNA was digested with 50 units BsgI in the presence of 80 µM S-adenosylmethionine according to the manufacturer's instructions. BsgI cuts 16 base pairs downstream from its recognition sequence leaving behind the randomly circular permuted *bla* gene library with a 2-nucleotide 3′ overhang. These two overhang nucleotides were degraded by treating the isolated PCR product with Klenow(exo+) in the presence of dNTPs to create the blunt insert.

One hundred ng of linear, blunt pC8BlaStop vector was ligated to this 100 ng insert. The 10-µl reaction was carried out at 22°C overnight (for 24 hours) using 30,000 Weiss units of T4 DNA ligase. After ethanol precipitation, 5% of the precipitated product was electroporated into 75 µl DH5α *E. coli* cells using 0.2 cm cuvettes. Eight separate electroporations were performed and combined. The total number of transformants in the cpBLA library was about 0.5×10^6^.

### Library characterization and selection

A frozen stock of the naïve library was diluted and plated on LB agar plates containing no antibiotics, 16 µg/ml ampicillin (Amp) or 250 µg/ml Amp. The plates were incubated overnight at room temperature. Plasmid DNA from inoculum prepared from randomly selected colonies were sequenced.

To select from genes that conferred increase resistance to β-lactam containing antibiotics, frozen stocks of the naïve library were diluted and plated on LB agar plates containing ampicillin, cefazolin, cephalothin or cefotaxime at concentrations at or above the MIC provided by *bla*. The plates were incubated overnight at 37°C and room temperature. Only on cefotaxime containing plates did any library members grow. The plasmid DNA was isolated and retransformed into DH5α *E.coli* to confirm that the reason for the elevated levels of resistance was plasmid-borne.

### Minimum inhibitory concentration determination

Minimum inhibitory concentrations (MICs) were determined by plating dilutions of overnight inoculums supplemented with varying concentrations of ampicillin, cefazolin, cephalothin or cefotaxime. Colonies that appeared on the LB plates after 24 hours incubation at room temperature were counted. The lowest concentration of the antibiotic required to prevent growth of at least 99% of the plated cells (compared to plates with no antibiotic) was taken to be the MIC.

## References

[1] Meister GE, Kanwar M, Ostermeier M, Lutz S, Bornscheuer U (2009). Circular permutation of proteins.. Protein Engineering Handbook: Wiley.

[2] Baird GS, Zacharias DA, Tsien RY (1999). Circular permutation and receptor insertion within green fluorescent proteins.. Proc Natl Acad Sci USA.

[3] Graf R, Schachman HK (1996). Random circular permutation of genes and expressed polypeptide chains: Application of the method to the catalytic chains of aspartate transcarbamoylase.. Proc Natl Acad Sci USA.

[4] Topell S, Hennecke J, Glockshuber R (1999). Circularly permuted variants of the green fluorescent protein.. FEBS Lett.

[5] Iwakura M, Nakamura T, Yamane C, Maki K (2000). Systematic circular permutation of an entire protein reveals essential folding elements.. Nat Struct Biol.

[6] Cheltsov AV, Barber MJ, Ferreira GC (2001). Circular permutation of 5-aminolevulinate synthase. Mapping the polypeptide chain to its function.. J Biol Chem.

[7] Qian Z, Lutz S (2005). Improving the catalytic activity of Candida antarctica lipase B by circular permutation.. J Am Chem Soc.

[8] Qian Z, Fields CJ, Lutz S (2007). Investigating the structural and functional consequences of circular permutation on lipase B from Candida antarctica.. Chembiochem.

[9] Reitinger S, Yu Y, Wicki J, Ludwiczek M, D'Angelo I (2010). Circular permutation of Bacillus circulans xylanase: a kinetic and structural study.. Biochemistry.

[10] Yu Y, Lutz S (2009). Improved triglyceride transesterification by circular permuted Candida antarctica lipase B.. Biotechnol Bioeng.

[11] Qian Z, Horton JR, Cheng X, Lutz S (2009). Structural redesign of lipase B from Candida antarctica by circular permutation and incremental truncation.. J Mol Biol.

[12] Guntas G, Mitchell SF, Ostermeier M (2004). A molecular switch created by in vitro recombination of nonhomologous genes.. Chem Biol.

[13] Ostermeier M (2005). Engineering allosteric protein switches by domain insertion.. Protein Engineering Design and Selection.

[14] Fisher JF, Mobashery S (2009). Three decades of the class A beta-lactamase acyl-enzyme.. Curr Protein Pept Sci.

[15] Hayes F, Hallet B, Cao Y (1997). Insertion mutagenesis as a tool in the modification of protein function. Extended substrate specificity conferred by pentapeptide insertions in the omega-loop of TEM-1 beta-lactamase.. J Biol Chem.

[16] Petrosino JF, Palzkill T (1996). Systematic mutagenesis of the active site omega loop of TEM-1 beta-lactamase.. J Bacteriol.

[17] Salverda ML, De Visser JA, Barlow M (2010). Natural evolution of TEM-1 beta-lactamase: experimental reconstruction and clinical relevance.. FEMS Microbiol Rev.

[18] Osuna J, Pérez-Blancas A, Soberón X (2002). Improving a circularly permuted TEM-1 β-lactmase by directed evolution.. Protein Eng.

[19] Guntas G, Mansell TJ, Kim JR, Ostermeier M (2005). Directed evolution of protein switches and their application to the creation of ligand-binding proteins.. Proc Natl Acad Sci USA.

[20] Bosley AD, Ostermeier M (2005). Mathematical expressions useful in the construction, description and evaluation of protein libraries.. Biomolecular Engineering.

[21] Minasov G, Wang X, Shoichet BK (2002). An ultrahigh resolution structure of TEM-1 beta-lactamase suggests a role for Glu166 as the general base in acylation.. J Am Chem Soc.

[22] Ostermeier M, Nixon AE, Shim JH, Benkovic SJ (1999). Combinatorial protein engineering by incremental truncation.. Proc Natl Acad Sci USA.

[23] Ostermeier M, Shim JH, Benkovic SJ (1999). A combinatorial approach to hybrid enzymes independent of DNA homology.. Nat Biotechnol.

